# A Proteomic Perspective on the Bacterial Adaptation to Cold: Integrating OMICs Data of the Psychrotrophic Bacterium *Exiguobacterium antarcticum* B7

**DOI:** 10.3390/proteomes5010009

**Published:** 2017-02-23

**Authors:** Rafael A. Baraúna, Dhara Y. Freitas, Juliana C. Pinheiro, Adriana R. C. Folador, Artur Silva

**Affiliations:** Laboratory of Genomics and Bioinformatics, Center of Genomics and Systems Biology, Institute of Biological Sciences, Federal University of Pará, Belém 66075-110, Brazil; dharayf@gmail.com (D.Y.F.); julianacrz24@gmail.com (J.C.P.); carneiroar@gmail.com (A.R.C.F.); asilva@ufpa.br (A.S.)

**Keywords:** genomic, transcriptomic, proteomic, *Exiguobacterium antarcticum* B7, 2DE

## Abstract

Since the publication of one of the first studies using 2D gel electrophoresis by Patrick H. O’Farrell in 1975, several other studies have used that method to evaluate cellular responses to different physicochemical variations. In environmental microbiology, bacterial adaptation to cold environments is a “hot topic” because of its application in biotechnological processes. As in other fields, gel-based and gel-free proteomic methods have been used to determine the molecular mechanisms of adaptation to cold of several psychrotrophic and psychrophilic bacterial species. In this review, we aim to describe and discuss these main molecular mechanisms of cold adaptation, referencing proteomic studies that have made significant contributions to our current knowledge in the area. Furthermore, we use *Exiguobacterium antarcticum* B7 as a model organism to present the importance of integrating genomic, transcriptomic, and proteomic data. This species has been isolated in Antarctica and previously studied at all three omic levels. The integration of these data permitted more robust conclusions about the mechanisms of bacterial adaptation to cold.

## 1. Introduction

One of the first omic studies performed and published was the 2D gel electrophoresis (2DE) of *Escherichia coli* cultures by Patrick H. O’Farrell in 1975 [1]. Thirteen years later, the development of two ion sources for mass spectrometry—matrix-assisted laser desorption/ionization (MALDI) and electrospray ionization (ESI)—allowed the identification of proteins extracted from 2DE spots [2,3]. Many years later, the development of next-generation sequencing (NGS) elevated the importance of omics sciences and led to the preparation of an essential database of gene sequences to assist proteomic approaches. Several studies in the environmental microbiology field have used 2DE and other proteomic techniques to answer questions about microbial adaptation to different environmental variables. In this context, bacterial adaptation to cold environments is a “hot topic” because of its application in biotechnological processes.

Several microorganisms have the ability to tolerate extreme environmental conditions, which may be essential for their survival [4]. Antarctica is one of the most extreme environments on the planet, and microbial habitats in this region include marine waters, air, snow, glacial ice, surface soils, and permafrost. Psychrophilic and psychrotrophic organisms that inhabit this polar region are constantly exposed to variations in temperature, desiccation, high or low levels of salinity or pH, prolonged periods in the absence of light during the winter, and high levels of ultraviolet (UV) radiation.

To cope with those environmental stresses and to survive and grow in low-temperature environments, those microorganisms exhibit several mechanisms of physiological adaptation, which are not ubiquitous in other bacteria. These mechanisms include (i) the increased fluidity of cell membranes; (ii) a reduced freezing point of the aqueous phase of the cytoplasm and stabilization of macromolecules; (iii) cellular responses to temperature decreases through cold shock and cold acclimation proteins (CSPs and CAPs, respectively); (iv) protection against reactive oxygen species (ROS) through catalases, peroxidases, superoxide dismutase, and oxidoreductases; (v) and the maintenance of catalytic efficiency in the cold [5].

The genus *Exiguobacterium* comprises species that have been isolated from several habitats with a wide temperature range (from −12 °C to 55 °C) including glacial ice, hot springs, the rhizosphere of plants, Siberian permafrost, and tropical and temperate soils [6]. This genus harbors psychrotrophic, mesophilic, and moderate thermophilic species and strains with biotechnological, industrial, bioremediation, and agricultural properties of interest [7]. Currently, there are four species of this genus whose genomes are completely sequenced including *Exiguobacterium antarcticum* B7 and draft genome sequences for other *Exiguobacterium* strains [8,9,10,11].

Recent works using *E. antarcticum* B7 demonstrated the importance of combining different molecular approaches to better understand bacterial adaptation to cold [12,13,14]. System biology analyses using the transcriptome data of *E. antarcticum* B7 identified a potential change in the metabolic pathway of fatty acids in response to cold [13]. Thus, genomic, transcriptomic, proteomic, and other omic technologies generate a large quantity of data that can be used and integrated to better formulate hypotheses about the mechanisms of microbial adaptation.

In this review, we aim to describe and discuss the main molecular modifications that occur in bacterial cells when exposed to low temperatures and the importance of omics technologies in this field. Furthermore, we use *Exiguobacterium antarcticum* B7 as a model organism to present the importance of integrating genomic, transcriptomic, and proteomic data to allow more robust conclusions about the mechanisms of bacterial adaptation to cold.

## 2. Mechanisms of Bacterial Adaptation to Cold

### 2.1. Chemical Modification of the Cellular Membrane

The cell membrane of prokaryotes becomes more rigid in low temperatures, and some chemical changes occur in the membrane fatty acids to prevent cellular damage. New lipid molecules are synthesized or modified to produce lipids with a low gel-liquid crystalline phase transition to maintain membrane fluidity [15]. The main changes observed in the membrane fatty acids include an increase in the number of unsaturations and methyl groups, a decrease in the chain length, and an increased rate of anteiso chemical ramifications compared to the iso ramifications [15]. This process of membrane adaptation is commonly termed homeoviscous adaptation [16]. Polyunsaturated fatty acids (PUFAs) have a much lower melting temperature compared to monounsaturated fatty acids. Thus, PUFAs are responsible to maintain membrane fluidity even in temperatures below 0 °C. The unsaturated branched-chain fatty acids are generated by anaerobic (de novo synthesis) or aerobic pathways (post-synthesis modification) [15].

An anaerobic pathway is commonly found in Gram-positive bacteria of the *Bacillales* order, where unsaturated branched-chain fatty acids are synthesized from simpler molecules such as acetyl-CoA [17]. First, acetyl-CoA is converted to malonyl-CoA by acetyl-CoA caboxylase and subsequently linked to an acyl carrier protein (ACP), forming malonyl-ACP. This molecule undergoes successive rounds of elongation of its fatty acid chain through a cyclic pathway whose reactions are catalyzed by enzymes encoded by the genes *fabF*, *fabG*, *fabI*, and *fabH*. The newly synthesized fatty acid molecule is then linked to glycerol-3-phosphate to form phosphatidic acid, which is a key intermediate molecule of all membrane glycerolipids [17].

In the aerobic pathway, the unsaturations are introduced directly into the membrane phospholipids by desaturase enzymes through dehydrogenation reactions. In *Bacillus subtilis*, the expression of Δ5-fatty acid desaturase is activated by a two-component system called DesR-DesK [18]. It has been suggested that a change in membrane fluidity caused by low temperatures result in conformational changes in DesK, triggering autokinase activity [19]. Once activated, DesK phosphorylates DesR, which binds to DNA, inducing the expression of desaturase genes [20,21].

Omic studies have allowed a better understanding of the microbial cold adaptation mechanisms through the identification of differentially expressed proteins. In a genomic study of *Colwellia psychrerytharea* the proteins involved in the synthesis, ramification, and cis-isomerization of polyunsaturated fatty acids were described [22]. Subsequently, the authors identified differentially expressed genes of polyunsaturated fatty acid synthases (*pfaC*, *pfaA*, and *pfaD*) [23]. To date, these synthase enzymes have been described only in marine bacteria [23,24]. In *Sphingopyxis alaskensis*, the enzymes involved in the de novo synthesis of fatty acids were described using quantitative proteomic approaches [25]. However, it was not possible to determine whether the bacterium produces new fatty acid chains or desaturates the existing membrane lipids. Recent studies have shown that two psychrotrophic species—*Exiguobacterium sibiricum* 255-15 and *Psychrobacter arcticus* 273-4—repress the expression of their genes associated with fatty acid biosynthesis while upregulating the genes associated with desaturation at low temperatures [9,26].

Interestingly, *E. sibiricum* 255-15 exhibited an increase in the expression of genes involved in peptidoglycan biosynthesis. An increase in cell wall density can protect bacteria against cell disruption that may be caused by ice formation and osmotic pressure at low temperatures [9]. The same behavior was observed in *Planococcus halocryophilus* Or1 [27]. In contrast, other studies have demonstrated that the species *P. arcticus* represses the expression of genes involved in peptidoglycan biosynthesis and enhances the expression of genes involved in the autolytic cleavage of the cell wall [26]. In *Sphingopyxis alaskensis,* a high abundance of proteins involved in cell wall biogenesis was described at 10 °C including a membrane structural lipoprotein OmpA which acts in the optimization of the structure and function of the membrane [25]. Recently, a transcriptomic analysis of *Listeria monocytogenes* cultivated under low temperatures and osmotic stress revealed the upregulation of genes associated with the biosynthesis of peptidoglycan and fatty acid molecules [28].

It is also important to note that the activity of membrane carriers is directly influenced by the lipidic state of the membrane [29]. The transport and diffusion through the membrane are also compromised at low temperatures. To balance this deficit, proteins of the transport system are upregulated. Despite the different mechanisms observed in the Bacteria domain, the molecular modifications at low temperatures have one single purpose: increase the number of membrane polyunsaturated branched-chain fatty acids to maintain membrane fluidity and the correct transport and diffusion of substances through this important biological barrier.

### 2.2. Cold-Adapted Enzymes

Microbial adaptation to extreme temperatures requires the evolution of enzymes to work with a high catalytic efficiency under these extreme conditions. Such extremophilic enzymes are valuable tools for studying the relationships between protein stability, dynamics, and function [30]. Low temperatures markedly reduce the k_cat_ of nearly all enzymatic reactions in a cell [31]. However, because this may not seem to be a significant barrier to microbial physiological processes, it is very clear that psychrophilic and psychrotrophic enzymes have adapted to efficiently operate at low temperatures. This enzymatic efficiency depends on the ratio between K_cat_/K_m_. K_cat_ measures how many substrate molecules are converted in products in a unit of time under optimal catalytic conditions. The K_cat_ constant is commonly called the “turnover number.” The constant K_m_ measures the substrate concentration that drives the reaction to half of its maximum velocity.

A high value of K_cat_ (fast turnover) and a low value of K_m_ (high affinity for a given substrate) increase the enzymatic efficiency. This enzymatic efficiency is directly dependent on the conformational dynamics of the enzyme. Using proteomic, molecular modeling, X-ray crystallography, and Nuclear Magnetic Resonance (NMR), it was observed that a low level of conformational stability allows cold-adapted enzymes to have high rates of enzymatic turnover at low temperatures [5,32,33]. These analyses led to the concept of “flexibility”, which describes the capacity of an enzyme to exhibit increased catalytic activity due to the loss of conformational stability. High flexibility occurs as a result of a reduction in the number of chemical interactions between the amino acids of the protein. This low molecular rigidity allows better complementarity between the active site and the substrate at a low energy cost. Many chemical factors of the enzyme contribute to increased catalysis in cold, including a decrease in the hydrophobicity of the protein core, a decrease in the number of aliphatic amino acids and protein residues forming salt bridges, and increased entropy. Not all of these characteristics are present in the same cold-adapted enzyme, but this list represents some of the changes observed by comparing psychrophilic enzymes to their mesophilic counterparts [34,35].

Amino acid composition seems to be an important characteristic to cold adaptation in several microorganisms. An α-amilase from the psychrophilic ciliated protozoon *Euplotes focardii* showed large modifications in amino acid composition when compared to an α-amilase of the mesophilic congeneric species *Euplotes crassus*. This modification consequently alters the types of intramolecular and surface chemical bonds [36]. Psychrophilic enzyme of *E. focardii* avoided charged, aromatic, and hydrophobic residues on its surface [36]. The genome of *Psychrobacter arcticus* 273-4 shows a statistically significant modification of amino acid composition compared to the mesophilic microorganisms, which can facilitate the flexibility of the proteins at low temperatures and consequently maintain cell viability in cold habitats [35]. Another example of altered amino acid composition is described in the genus *Vibrionaceae*. The psychrophilic species of the genus have proteins with a reduced number of proline residues [37]. Proline decreases the flexibility of the protein due to the rigidity of its nitrogen–carbon bond [38]. Thus, proline substitution in psychrophilic proteins increases flexibility of the molecule and consequently decreases the energy required to interact with the substrate. Arginine is also considered an amino acid that promotes structural rigidity since it forms salt bridges and hydrogen bonds with side chains of the protein structure [39]. A low amount of arginine has been observed in a thermolysin of the psychrophilic *Antarctic bacterium* [39].

### 2.3. Cold Shock and Cold Acclimation Proteins

One of the most prominent responses of microorganisms to cold environments is the expression of cold shock or cold acclimation proteins. It is important to note that psychrophilic and psychrotrophic as well as mesophilic and thermophilic microorganisms express cold shock proteins to neutralize the effects of temperature reduction. A cold shock response occurs when the microorganism is transferred from an optimal growth temperature to a cold temperature, triggering an immediate and transient molecular response. However, the acclimation process occurs when the bacteria remain exposed to cold for a long period, leading to a late and continuous molecular response [40]. CSPs are expressed by homologous genes that exhibit RNA chaperone activity and thus act to destabilize secondary structures of RNA erroneously formed due to exposure to cold. The activity of these proteins maintains the correct flux of the transcription and translation process in prokaryotes [41].

The first bacterial cold shock protein reported was CspA of *Escherichia coli* [42]. Subsequently, several other CSPs were described in a large range of Gram-positive and Gram-negative bacteria. In *E. coli*, cold shock proteins can be divided into two major groups: I and II. CSPs belonging to group I (CspA, CspB, CspG, CspI, CsdA, RbfA, NusA, and PNPase) are drastically induced at low temperatures compared to the CSPs of group II (RecA, IF-2, H-NS, GyrA, Hsc66, and HscB). CspA, CspB, CspG, and CspI act as RNA chaperones [41]. After cold shock, the expression of CSPs of group I is dramatically decreased while other proteins are expressed during the acclimation phase to maintain cell function. CsdA is a DEAD-box RNA helicase that increases septation, resulting in the formation of coccobacilli shape at low temperatures [43]. CsdA also acts as a RNA chaperone [43]. RbfA is a ribosome binding factor that is involved in ribosome maturation at cold temperatures [44]. Finally, PNPase is an enzyme that catalyzes the phosphorolysis of single-stranded polyribonucleotides and is the major factor responsible for the reduction of CSPs in bacterial cells after cold shock response [45].

Currently, several studies have reported other molecular functions of CSP homologues such as osmotic balance, protection to oxidative stress, starvation, and other types of stress, showing that this protein family has a greater importance than previously thought to the process of microbial adaptation to extreme conditions [46].

### 2.4. Other Important Aspects

In addition to the mechanisms of cold adaptation mentioned above, the production of carotenoids markedly contributes to bacterial survival in cold environments. Carotenoids are tetraterpenoids, pigments found naturally occurring in microorganisms, plants, and even animals. Carotenoids are synthesized by several species of bacteria, algae, and fungi in response to several environment stresses [47]. Prokaryotic organisms that produce carotenoids have been summarized by Takano and colleagues [48]. However, since 2006 several carotenoid-producing bacterial species were discovered in cold environments [49]. Carotenoids are detected in the membrane of psychrophilic [49], psychrotrophic [27], and mesophilic [50] bacterial species. The high frequency of pigment production in strains isolated from cold environments suggests that these pigments play an important role in the adaptation to this ecological niche [51]. At low temperatures, the production of polar carotenoids suppresses the production of non-polar carotenoids. This chemical modification was observed in *Arthrobacter agilis*, *Sphingobacterium antarcticus*, and *Micrococcus roseus* [50,52,53].

In addition, carotenoids protect free-living bacteria from high levels of UV radiation and promote resistance to cellular oxidative stress [49]. Several genes involved in carotenoid biosynthesis, such as *idi*, *crtE*, *crtB*, *crtI*, *crtEB*, *crtYe*, and *crtYf*, were described in bacterial species of the *Arthrobacter* genus isolated from Antarctic soils [49]. Recently, the Prokaryotic Carotenoid Database (ProCarDB) was created by using 304 unique carotenoids synthesized through 50 biosynthetic pathways distributed in 611 prokaryotes [54].

**Figure 1:**
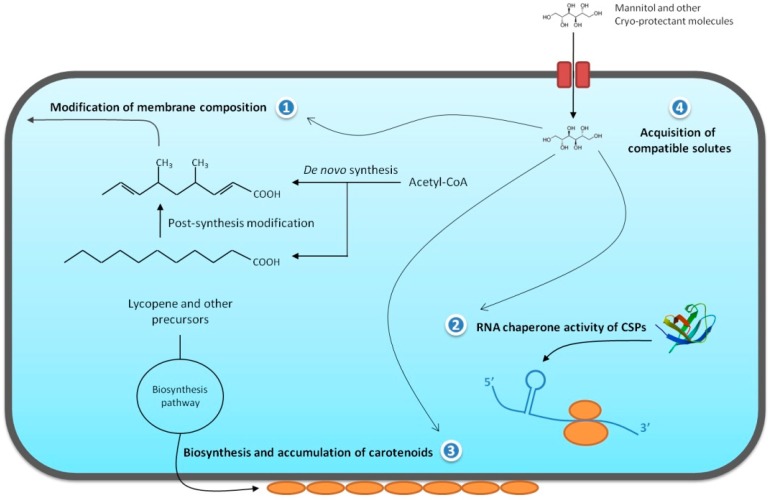
Representation of the main molecular modifications presented by bacterial cells during cold adaptation. Four adaptations are presented: (1) production of unsaturated branched-chain fatty acids to maintain membrane fluidity; (2) destabilization of adverse RNA structures by cold shock proteins; (3) production of carotenoids to assist in the maintenance of membrane fluidity and prevent cell damage by UV radiation; and (4) transport of compatible solutes such as mannitol to stabilize the cytoplasmic environment and prevent ice formation.

Additionally, thermal stress affects the osmotic balance of the microbial cell, resulting in a large efflux of cytoplasmatic water. Therefore, to prevent water loss and intracellular ice formation, bacterial cells accumulate compatible solutes in the cell cytoplasm. Examples of such cryo-protectant molecules are glucose, trehalose, glycogen, fructose, alanine, betaine, mannitol, and glycerol. These substances also prevent protein aggregation by stabilizing cytoplasmic macromolecules [55]. Figure 1 summarizes the main molecular modifications that occur in bacterial cells adapted to low temperatures, as described above.

## 3. *Exiguobacterium antarcticum* B7 as a Model Organism for Studies of Cold Adaptation

**Figure 2:**
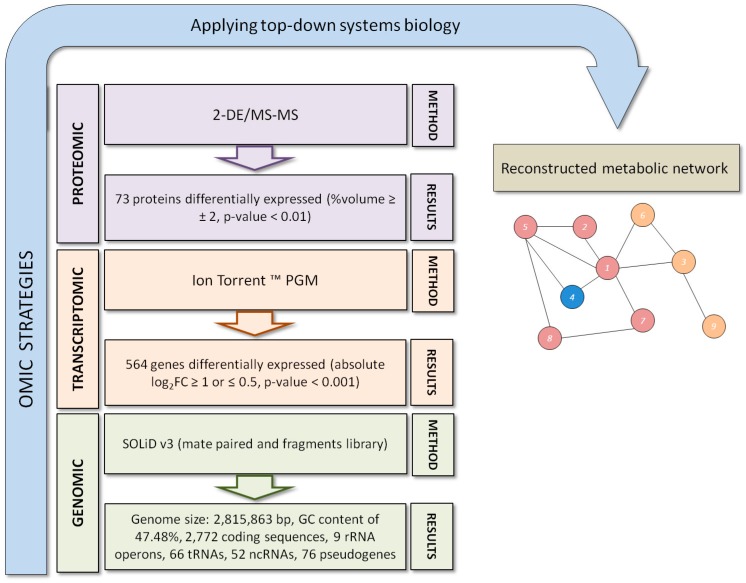
Flowchart showing the omics strategies used to study cold adaptation of *E. antarcticum* B7. Each omic analysis contains the method used and the results achieved. This large amount of data can then be used to reconstruct metabolic models using top-down approaches of systems biology.

*Exiguobacterium antarcticum* B7 is a psychrotrophic bacterium isolated from a biofilm formed in the sediment of Lake Ginger, Antarctic Peninsula [8]. Its optimal growth temperature is 37 °C, and its minimal growth temperature is −2 °C. *E. antarcticum* B7 has bacillary morphology that may change depending on the physicochemical conditions of the environment. Its genome was sequenced using NGS, and its gene expression at low temperatures was evaluated using transcriptomic and proteomic techniques [14]. Figure 2 summarizes the omic analyses performed and shows the methods and results obtained. In this approach, genomic analysis is the starting point for generating a large quantity of data that is subsequently used as the basis for the validation of experimental models through transcriptomics and proteomics (Figure 2). Subsequently, the data set generated by these high-throughput methods can be used in top-down models of systems biology, as discussed and proposed by Bernhard Palsson [56] after the emergence of the first NGS technologies in 2002. Recently, the metabolic pathway for the de novo biosynthesis of fatty acids in *E. antarcticum* B7 was reconstructed using constraint-based approaches [13]. Applying the log_2_FC (log base 2 Fold Change) of the transcriptome in the calculated model, the fluxome was modified and the metabolic pathway of *E. antarcticum* B7 started to produce short-chain fatty acids. This metabolic behavior has been experimentally documented for other cold-adapted bacteria [23,25].

**Figure A1:**
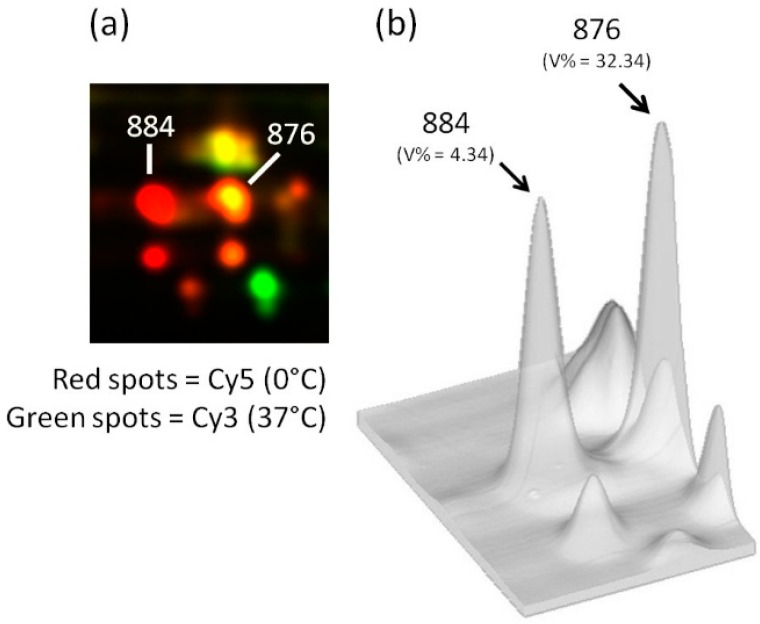
(**a**) Fraction of a Differential Gel Electrophoresis (DIGE) comparing the proteome of *E. antarcticum* grown at 37 °C (green spots) and 0 °C (red spots). Each spot is indicated by its ID; (**b**) Three-dimensional view of the spots with their respective values of volume percentage.

A total of 564 genes of *E. antarcticum* B7 were differentially expressed in cold. Gel-based proteomic analyses described 73 differentially expressed proteins [14]. Genes of *E. antarcticum* B7 that are involved in the five adaptive pathways described above are listed in Table 1. Interestingly, two cold shock proteins were downregulated in the cold (Csp5 and Csp6) (Table 1). These two proteins were not detected in the proteomic analysis. However, the other four CSPs were upregulated at 0 °C, and the proteomic results showed that Csp1 was 32-fold more expressed at low temperatures [14]. Thus, Csp1 is the main CPS of *E. antarcticum* B7. Additionally, Csp1 was detected in four different spots of the gel, two of which presented an interesting pattern of pI modification. These two spots have the same molecular weight and different pI values (Figure A1 in Appendix A). The results suggest a possible post-translational modification of CPSs at low temperatures such as phosphorylation. One of these proteoforms of Csp1 (spot 884) apparently appears only at 0 °C (Figure A1 in Appendix A).

Clearly, transcriptomic allowed a more embracing analysis of gene expression in *E. antarcticum* when compared to other omic approaches. However, transcriptomics has analytical limitations such as multi-mapping reads which may cause bias in the calculation of gene expression for homologous genes such as *csp* [57]. In this case, proteomics allowed the identification of the main CSP used by *E. antarcticum* during cold response. The other proteins identified belong to metabolic pathways commonly described in gel-based proteomic analyzes such as oxidative stress, heat shock proteins, and cellular respiration [58]. Those findings such as genomic signature, gene expression pattern, CSP proteoform identification, and reconstruction of metabolic networks, could only be achieved by using all levels of omic analyses, which emphasizes the need to integrate the data of these high throughput methods.

## 4. Conclusions and Future Perspectives

By observing the different metabolic behavior described in this review, it can be noted that different psychrotrophic and psychrophilic species have ecologically converged to adapt to low-temperature environments via different biological methods. Different proteomic and other omic approaches were used to achieve our current knowledge on microbial adaptation to cold. The ecological relationships between microorganisms living in cold environments have also been analyzed by metaproteomics [59].

The most recent proteomic methods based on liquid chromatography coupled with mass spectrometry (LC-MS/MS) can generate a large amount of data that can assist us in understanding important aspects of bacterial adaptation to cold. Methods such as selected reaction monitoring (SRM) in targeted-MS proteomic are now being used in the field of microbiology [60]. SRM is better applied to microorganisms that have been previously analyzed using high-throughput techniques.

Genomics has now reached a high level of sensitivity, precision, and accuracy in their analyses. Consequently, transcriptomics and proteomics methods have tended to evolve to generate large quantities of data with increased reliability. Rapid technological evolution has led to the development of sub-omic areas that will permit the analyses of microbial adaptation to different environments through a holistic perspective (e.g., surfomics is based on methods for rapid identification of cell surface proteins) [61]. Finally, bioinformatics is a strategic area for the development and biotechnological application of omics sciences, especially proteomics.

## Figures and Tables

**Table 1:** *E. antarcticum* B7 genes involved in metabolic pathways of cold adaptation. Next to the name of the genes are the Log_2_FC values and *p*-values of the transcriptome assays published by Dall’Agnol and colleagues [40]. Pseudogenes are identified in parentheses.

Genes	Log_2_FC	*p*-Value	Genes	Log_2_FC	*p*-Value
Cold shock proteins			De novo synthesis of fatty acids		
*csp1*	1.94	0	*accA*	0.45	0.01
*csp2*	2.16	5.43 × 10^−194^	*accB*	−0.05	0.01
*csp3*	2.30	0	*accC*	−0.56	5.68 × 10^−27^
*csp4*	2.46	0	*accD*	−0.28	7.55 × 10^−4^
*csp5*	−1.06	3.73 × 10^−35^	*fapR*	0.58	4.80 × 10^−21^
*csp6*	−1.28	4.41 × 10^−194^	*plsX*	0.77	2.31 × 10^−46^
Desaturation of membrane fatty acids			*fabD*	0.94	2.92 × 10^−85^
*desK*	7.03	8.15 × 10^−16^	*fabG*	0.85	8.42 × 10^−63^
*desR*	−0.48	9.37 × 10^−8^	*fabH1*	0.85	2.67 × 10^−26^
Transport of compatible solutes			*fabF*	0.69	3.46 × 10^−16^
*opuCA*	3.17	1.99 × 10^−62^	*fabI*	−1.74	0
*opuCC*	1.61	3.06 × 10^−29^	*plsC*	−0.82	1.28 × 10^−6^
*opuE*	3.70	4.26 × 10^−16^	Carotenoid biosynthesis		
*opuCD*	−2.72	8.67 × 10^−24^	*crtI (pseudo)*	3.94	4.65 × 10^−43^
*opuBA*	−0.52	6.11 × 10^−4^	*yisP1 (pseudo)*	−1.33	3.09 × 10^−41^
			*yisP2*	0.75	2.16 × 10^−7^
